# Changes in Serum Thiol-Disulphide Homeostasis in Sheep with Gastrointestinal Nematodes

**DOI:** 10.3390/ani11102856

**Published:** 2021-09-30

**Authors:** Elizabeth Moreira dos Santos Schmidt, Daniele Floriano Fachiolli, Raphaela Moreira de Oliveira, Fabiana Alves Almeida, Cristiano Magalhães Pariz, Paulo Roberto de Lima Meirelles, Ciniro Costa, Asta Tvarijonaviciute, Ozcan Erel, Salim Neselioglu, Jose Joaquin Ceron, Camila Peres Rubio

**Affiliations:** 1 Department of Veterinary Clinical Sciences, School of Veterinary Medicine and Animal Science, Campus of Botucatu, São Paulo State University (FMVZ-UNESP), Botucatu 18618-681, Brazil; elizabeth.schmidt@unesp.br (E.M.d.S.S.); raphaelamoreira7@gmail.com (R.M.d.O.); 2 Faculty of Veterinary Medicine, University Center of the Federal District-UDF, Brasilia 70390-030, Brazil; 3 Department of Animal Nutrition and Breeding, School of Veterinary Medicine and Animal Science, Campus of Botucatu, São Paulo State University (FMVZ-UNESP), Botucatu 18618-681, Brazil; daniele.fachiolli@unesp.br (D.F.F.); cmpzoo@gmail.com (C.M.P.); paulo.meirelles@unesp.br (P.R.d.L.M.); ciniro.costa@unesp.br (C.C.); 4 Department of Parasitology, Biosciences Institute, Campus of Botucatu, São Paulo State University (FMVZ-UNESP), Botucatu 18618-681, Brazil; faalvesalmeida@yahoo.com.br; 5 Interdisciplinary Laboratory of Clinical Analysis of the University of Murcia (Interlab-UMU), Regional Campus of International Excellence ‘Campus Mare Nostrum’, University of Murcia, Espinardo, 30100 Murcia, Spain; asta@um.es (A.T.); jjceron@um.es (J.J.C.); 6 Department of Biochemistry, Faculty of Medicine, Ankara Yildirim Beyazit University, Ankara City Hospital, Ankara 06800, Turkey; erelozcan@gmail.com (O.E.); salim_neselioglu@hotmail.com (S.N.)

**Keywords:** antioxidant, closantel, *Haemonchus*, integrated crop-livestock, oxidative stress, ruminants

## Abstract

**Simple Summary:**

Parasitism with gastrointestinal nematodes represents a significant risk to the health of livestock populations. Besides the local oxidative damage caused by the parasite, the host reacts by increasing the production of oxidants. The study of thiol-disulphide homeostasis can be of help in the evaluation of the oxidative status of sheep during this type of parasitism. In this study, the thiol-disulphide homeostasis, together with other biomarkers of oxidative stress and inflammation, were assessed in the serum of lambs infected with gastrointestinal nematodes and were evaluated after 70 days of integrated crop-livestock system and anthelmintic treatment. This study showed that the thiol-disulphide balance was impaired in the infected lambs and the changes were correlated with the parasite load, which therefore could indicate their potential use as a tool to evaluate and monitor the disease in sheep.

**Abstract:**

This work aimed to evaluate the thiol-disulphide homeostasis in serum of lambs naturally infected by gastrointestinal nematodes presenting different levels of parasite load indirectly indicated by faecal worm egg counts (EPG). Furthermore, the possible changes in the thiol-disulphide dynamic after different procedures to reduce the parasitic charge, such as the integrated crop-livestock system or anthelmintic treatment, were assessed. The results were compared with a panel of various oxidative stress and inflammatory biomarkers. The lambs were divided into three groups: animals highly infected (EPG higher than 5000) and packed cell volume (PCV) lower than 24% (G1); animals highly infected (EPG higher than 5000) and normal PCV (>24%) (G2); and animals presenting EPG lower than 5000 and normal PCV (>24%) (G3). The highly infected lambs (G1 and G2) showed lower total thiol (TT) and native thiol (SH) (*p* ≤ 0.01) than those from G3. After treatment, TT and SH increased significantly in all groups (*p* ≤ 0.01), and the disulphide (SS)/TT and SS/SH ratios decreased significantly (*p* < 0.01) in G1 and G2. These results show that the thiol-disulphide balance was impaired in lambs infected by gastrointestinal nematodes and that it could be potentially used as a biomarker to monitor this disease.

## 1. Introduction

Parasitism with gastrointestinal nematodes represents the main risk to livestock populations’ health, welfare, and productivity throughout the world. Particularly in sheep, it is an important cause of the drop in production [1,2]. This parasitic gastroenteritis is associated with different species of nematodes such as *Haemonchus contortus*, *Teladorsagia circumcincta*, and *Trichostrongylus axei* that mainly act in the abomasum; *Trichostrongylus colubriformis*, *Cooperia curticei*, and *Nematodirus spathiger* in the small intestine; and *Oesophagostomum venulosum* in the large intestine [3]. In tropical areas, the most predominant are *H. contortus* and *T. colubriformis* [4], which are considered the most important in small ruminants [5,6].

Almost all sheep are infected with one or more of these nematodes in field conditions. The infection occurs when animals are grazing contaminated pasture, and the mix of larvae species (third-stage) ingested infects both the abomasum and intestine. The worm completes its development in adults in the gastrointestinal tract in approximately three weeks, and some species such as *H. contortus* may survive over a year inside a host sheep [7]. The gastrointestinal nematode parasitism may result in severe clinical disease, particularly related to the hematophagous *H. contortus* [8]. However, the disease is rarely due to just one parasitic species but rather to the cumulative effects of mixed parasitism [3]. Furthermore, the intensity of infection and clinical signs related to the disease could vary significantly depending on factors such as the severity of parasitism in the gastrointestinal tract, the general health and immunological status of the animal, type of climate and pasture, management, and diet [9]. Faecal worm egg counts (EPG) associated with the packed cell volume (PCV) are basic approaches that help to determine the severity of the infection [4,10,11].

Currently, the integrated crop-livestock (ICL) system, consisting of a single farm or areas managed by integrating agricultural crops and livestock, is increasingly used to eliminate the free-living stages of gastrointestinal nematodes from the pasture [12]. However, in situations where the high parasitic load is associated with severe clinical signs, treatment using chemical compounds with a broad spectrum of action is still used [13].

The inflammation in the abomasal mucosa due to the parasite attachment is an initial response to control the parasitic load. In addition, the parasites lead to local oxidative damage during the digestion of host tissue [14]. Furthermore, in an attempt to damage the parasite, the host reacts by increasing the production of reactive oxygen species (ROS), which, depending on the antioxidant system’s capacity, could produce a state of oxidative stress [15]. The local production and expression of oxidants and antioxidants in the gastrointestinal tract following a nematode challenge have been reported in sheep [16]. In addition, changes in serum biomarkers of oxidative stress have been described in lambs and sheep experimentally or naturally infected by gastrointestinal nematodes such as *H. contortus* [17,18,19,20,21].

The study of thiol-disulphide homeostasis, which involves the analysis of serum total thiol (TT), native thiol (SH), and disulphide (SS) concentrations, is gaining interest in human medicine to evaluate oxidative status in different diseases [22,23,24,25]. Previous studies suggested that thiol-disulphide homeostasis can be used as a marker of oxidative stress in sheep during sarcoptic mange infection [26]; however, to the author’s knowledge, no information about the evaluation of these biomarkers in gastrointestinal parasitism in sheep has been reported.

The main objective of this study was to determine serum levels of thiol-disulphide in lambs naturally infected by gastrointestinal nematodes presenting different levels of parasite load and clinical signs of the disease, in order to evaluate whether thiol-disulphide balance could be used as a new marker of oxidative stress in this disease. Furthermore, the second objective of this work was to evaluate the possible changes in thiol-disulphide levels after the reduction of the EPG and consequently the parasite burden, by different procedures such as ICL and anthelmintic treatment. 

## 2. Materials and Methods

### 2.1. Animals

Forty-one lambs of the breed Corriedale, aged approximately 90 days and originally from southern Brazil, were included in this study. The animals were raised under commercial and extensive breeding conditions, and before starting the experimental period, they received vitamin supplementation (Vitagold^®^) and a dose of a prophylactic vaccine for botulism, symptomatic carbuncle, gaseous gangrene, enterotoxaemia, and sudden death of ruminants (Poli-Star^®^, Vallée). They were then introduced in the ICL system, and during the first day of study (June 28), blood and faecal samples were obtained. Three groups were defined according to the cut-off value of 5000 EPG in agreement with a previous study [27] and the presence of decreased PCV to the species (reference intervals for the species is 24 to 50% [28]). The first group (G1) was integrated by 15 animals presenting EPG higher than 5000 and PCV lower than 24%; group 2 (G2) was integrated by 14 animals presenting EPG higher than 5000 and normal PCV (>24%), and group 3 (G3) was comprised by those lambs presenting EPG lower than 5000 and normal PCV (>24% (*n* = 12)). 

The study was developed in the experimental farm of the School of Veterinary Medicine and Animal Science, São Paulo State University, Botucatu, Brazil, in an experimental area in which an ICL system was planned to be implanted and conducted during winter, from 28 June to 6 September 2017, for 70 days as indicated previously [12]. Tropical conditions characterized the climate with dry winter. Daily at 6:00 a.m., all lambs were released from the shed to the paddocks where they grazed and had water ad libitum from drinking fountains. During the day, they were maintained in a pasture of black oats (*Avena strigosa*), overrun in the area after a harvest of maise intercropped with palisade grass (*Urochloa brizantha*, common name Marandu) to produce silage in an ICL system, which was offered to the animals. In the late afternoon, the lambs were housed in a covered shed where they were feed with maise silage and concentrate. Maise with marandu palisade grass was sown in the same area in the summer, and mechanical harvesting for ensilage was conducted at the beginning of the autumn. The roughage:concentrate ratio was 30:70. The diet of the lambs was formulated via the computer program Small Ruminant Nutrition System (SRNS) based on the Cornell Net Carbohydrate and Protein System (CNCPS) for sheep [29].

As clinical anaemia is the determinant for treating the animals with anthelmintic drugs for gastrointestinal nematodes [30], the animals presenting a PCV lower than 24% (G1) received a treatment consisting of closantel, 10 mg/Kg live weight (Diantel^®^, RS-Brazil) [12]. The other two groups of sheep were just maintained in the ICL system following previous recommendations [12,31]. 

The Ethics Committee of the School of Veterinary Medicine and Animal Sciences (CEUA 0152/2017), São Paulo State University (FMVZ-UNESP), Brazil, approved the study following current legislation on animal protection.

### 2.2. Sampling

Faeces and blood samples were taken from each animal on the first day of the initiation of the ICL system and 70 days after. The sampling was made at 06:30 a.m. while they were still fasting and in their shed. Blood samples were collected from the jugular vein and placed in tubes with EDTA (BD Vacutainer^®^ K2 EDTA Blood Collection Tube; Becton, Dickinson and Company, Franklin Lakes, NJ, USA) for haematology analysis and with a vacuum system and gel separator (BD Vacutainer^®^ Blood Collection Tube; Becton, Dickinson and Company, NJ, USA) in others to obtain serum. The vacuum tubes were centrifuged at 1500× *g* for 5 min and the serum obtained was stored frozen (−80 °C) in Eppendorf tubes (Eppendorf, Hamburg, Germany) until analysis. Faeces were collected directly from the rectum, stored in labelled plastic bags at 4 °C, and taken to the Veterinary Helminthology Laboratory at the UNESP—Institute of Biosciences, Campus of Botucatu, Brazil, where they were analysed.

### 2.3. Faecal Examination

The EPG was performed using the modified McMaster technique [32] following the specifications for small ruminants (2 g of faeces diluted in 58 mL of a hypersaturated solution of NaCl), where each egg counted represented 100 eggs/g of faeces. The larval culture [33] was done in a pool of samples from all the animals for identifying genera of third-stage infective larvae (L3) [34].

### 2.4. Analysis

#### 2.4.1. Blood Samples

The PCV was determined by the microhematocrit method [28].

#### 2.4.2. Thiol-Disulphide Homeostasis

Serum TT, SH and SS concentrations were determined by using a colorimetric method [35]. In brief, dynamic SS bonds in the sample were reduced to functional thiol groups (SH) by sodium borohydride (NaBH_4_). The excess of NaBH_4_ was completely removed by formaldehyde, preventing the extra reduction of the 5,5′-dithiobis-2-nitrobenzoic acid (DTNB) and further reduction of the SS formed, which was produced after the DTNB reaction. The TT content of the sample was measured using a modified Ellman reagent. The SH content was subtracted from the TT content, and half of the obtained difference gave the SS bond amount. In addition, the SS/TT, SS/SH, and SH/TT ratios were calculated.

The assays were performed using the Olympus AU400 (AU400 Automatic Chemistry Analyser, Olympus Europe GmbH, Hamburg, Germany) and showed lower than 15% imprecision.

#### 2.4.3. Oxidative Status Biomarkers

The results of the thiol-disulphide homeostasis were compared with a comprehensive panel of various automatic antioxidant (cupric reducing antioxidant capacity (CUPRAC), ferric reducing ability of plasma (FRAP), Trolox equivalent antioxidant capacity (TEAC), and uric acid) and oxidant (total oxidant status (TOS), ferrous oxidation-xylenol orange (FOX), reactive oxygen metabolites derived compounds (d-ROMs), and advanced oxidation protein products (AOPP)) biomarkers usually included in the profiles to evaluate oxidative stress.

Briefly, the CUPRAC was measured following an assay previously described [36]. It is based on reducing cupric-bathocuproinedisulfonic acid to a cuprous-bathocuproinedisulfonic acid by the sample. The determination of TEAC was based on the reduction of the radical 2,2′-azino-bis(3-ethylbenzthiazoline-6-sulfonic acid) (ABTS) pre-generated enzymatically with horseradish peroxidase (HRP) [37]. The measurement of FRAP was based on reducing the ferric-tripyridyltriazine to ferrous-tripyridyltriazine by the non-enzymatic antioxidants present in the sample [38]. Uric acid was determined by a commercially available spectrophotometric method (Uric Acid reagent OSR6698 Beckman Coulter AU analysers, Nyon, Switzerland). Serum PON1 was determined using a previously assay based on p-nitrophenyl acetate as substrate [39].

Serum TOS was measured by a method [40] based on the oxidation of the ferrous ion–o-dianisidine complex. The FOX assay was determined based on the automatic determination of the ferrous oxidation by xylenol orange [41]. The d-ROMs assay was based on monitoring the *N,N*-Diethyl-*p*-phenylenediamine radical cation concentration as previously published [42]. The indirect evaluation of oxidative damage to proteins was done by the measurement of AOPP, which was based on the determination of di-tyrosine containing cross-linked proteins and oxidatively modified albumin as previously described [43].

All assays were performed using the Olympus AU400 and showed imprecision lower than 15%.

#### 2.4.4. Inflammatory Biomarkers

Haptoglobin (Hp) was determined by a colorimetric method (kit haptoglobin Tridelta phase range, Tridelta Development Ltd., Bray, Ireland) commercially available. Butyrylcholinesterase (BChE) was analysed according to a previous assay based on butyrylthiocholine iodide as substrate [44].

Total protein and albumin concentrations were measured following the instructions of the manufacturer using Olympus commercial kits (Total protein OSR 6132, Albumin OSR 6102, Olympus Life and Material Science Europe GmbH, Hamburg, Germany). Globulin concentrations were calculated by the difference between total proteins and albumin concentrations. In addition, the albumin/globulin (AGR) ratio was calculated (AGR = Albumin/(Total protein − Albumin).

#### 2.4.5. Statistical Analysis

Data analyses were performed using Excel (Microsoft, Redmond, WA, USA) and Graph Pad Software Inc. (GraphPad Prism, version 6 for Windows, Graph Pad Software Inc., San Diego, CA, USA). A value of *p* < 0.05 was considered significant. Descriptive statistics were used to describe the basic features. The different biomarker concentrations were evaluated for normality of distribution using the Shapiro–Wilk normality test. All results met the normal distribution criteria except for EPG values, which were log-transformed prior to the analysis. Data are presented as means ± standard deviation in bar graphs. Two-way analysis of variance (ANOVA) mixed model of repeated measured tracked by Sidak’s multiple comparison test was used to evaluate the results over time from each group of animals. Two-way ANOVA followed by the Tukey post hoc multiple comparison analysis was performed to compare the results of each biomarker over time (days 0 and 70) comparing between the groups. Spearman’s correlation coefficients were calculated to assess the relationship between all biomarkers and the EPG values.

## 3. Results

### 3.1. Animals

**Figure 1:**
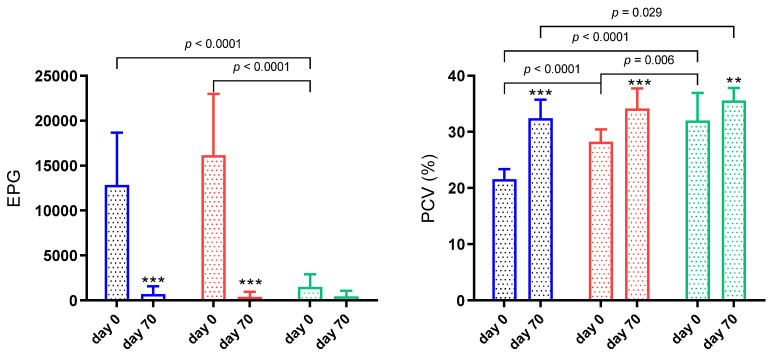
Faecal worm egg counts (EPG) and packed cell volume (PCV) in sheep naturally infected by gastrointestinal nematodes included in an integrated crop-livestock (ICL) system for 70 days. G1 (blue bar): EPG higher than 5000 and PCV lower than 24%; G2 (red bar): EPG higher than 5000 and PCV higher than 24%; G3 (green bar): EPG lower than 5000 and PCV higher than 24%. Asterisks indicate significantly different from day 0. ** *p* < 0.01; *** *p* < 0.001.

Results of EPG and PCV in each group of sheep infected by gastrointestinal nematode are shown in Figure 1. When groups were compared at day 0, G1 and G2 presented higher EPG counts (*p* < 0.0001) than G3. On day 0, the third larval stage of *Haemonchus* and *Trichostrongylus* were identified in 94.5% and 5.5% of the animals, respectively. On day 70, *Haemonchus* was identified in 73.1%, *Trichostrongylus* in 23% and *Cooperia* in 3.8% of the animals. 

Due to the high percentage of *Haemonchus* at the beginning of the trial and the low PCV, the lambs from G1 received anthelmintic treatment with closantel, 10 mg/Kg live weight (Diantel^®^, RS-Brazil) [12] two days after the first sampling. The rest of the animals were not treated since it was expected that the ICL system would reduce their parasitic burden.

After 70 days of being introduced in the ICL system and the closantel treatment, the EPG in G1 decreased significantly (*p* < 0.001). In the same way, the EPG in G2 decreased significantly at the end of the trial (*p* < 0.001). Regarding G3, although no statistical difference was observed (*p* > 0.05), the EPG values decreased compared to day 0 (Figure 1).

When groups were compared at the beginning of the trial, the PCV was different between all of them (*p* < 0.05). The PCV increased at the end of the experiment in all groups compared with the first sampling (*p* < 0.01) (Figure 1).

A significant interaction between time and group was observed in EPG (*p* < 0.0001) and PCV (*p* = 0.0002).

### 3.2. Thiol-Disulphide Homeostasis

**Figure 2:**
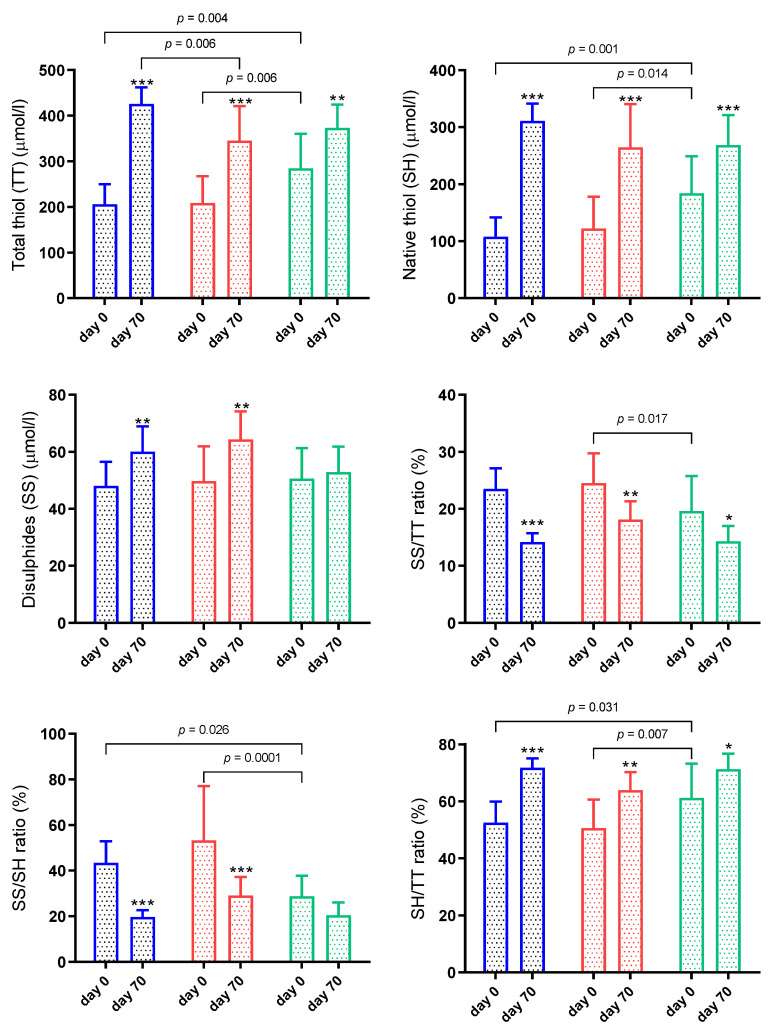
Results for thiol-disulphide homeostasis in sheep naturally infected by gastrointestinal nematodes. G1 (blue bar): faecal worm egg counts (EPG) higher than 5000 and packed cell volume (PCV) lower than 24%; G2 (red bar): EPG higher than 5000 and PCV higher than 24%; G3 (green bar): EPG lower than 5000 and PCV higher than 24%. Asterisks indicate significantly different from day 0. * *p* < 0.05; ** *p* < 0.01; *** *p* < 0.001.

The results for the thiol-disulphide homeostasis system in sheep naturally infected by gastrointestinal nematodes are shown in Figure 2. On day 0, significant differences in thiol-disulphide analytes were found between the groups of sheep, with G1 and G2 showing lower values of TT and SH than G3 (*p* ≤ 0.01) (Figure 2). The SS/TT and SS/SH ratios were significantly higher (*p* < 0.05) in G2, and in G1 and G2, respectively, than G3 on the first day of the trial. On the same day, G1 and G2 also exhibited a lower SH/TT ratio than G3 (*p* < 0.05). 

When biomarkers were compared before and after treatment, the TT and SH increased significantly (*p* < 0.01) on day 70 in G1, G2 and G3. Higher SS levels (*p* < 0.01) were observed in G1 and G2 at the end of the trial. The SS/TT ratio in all groups and the SS/SH ratio in G1 and G2 were decreased (*p* < 0.05) on day 70 compared to day 0. On the other hand, the SH/TT ratio in G1, G2 and G3 were significantly higher (*p* < 0.05) at the end of the trial (Figure 2).

Significant interaction between time and group was observed in TT (*p* = 0.001) and SH (*p* = 0.001). On the other hand, there was no significant interaction between time and group in SS (*p* = 0.074), SS/TT (*p* = 0.244), SS/SH (*p* = 0.088), SH/TT (*p* = 0.169).

### 3.3. Antioxidant Biomarkers

**Figure 3:**
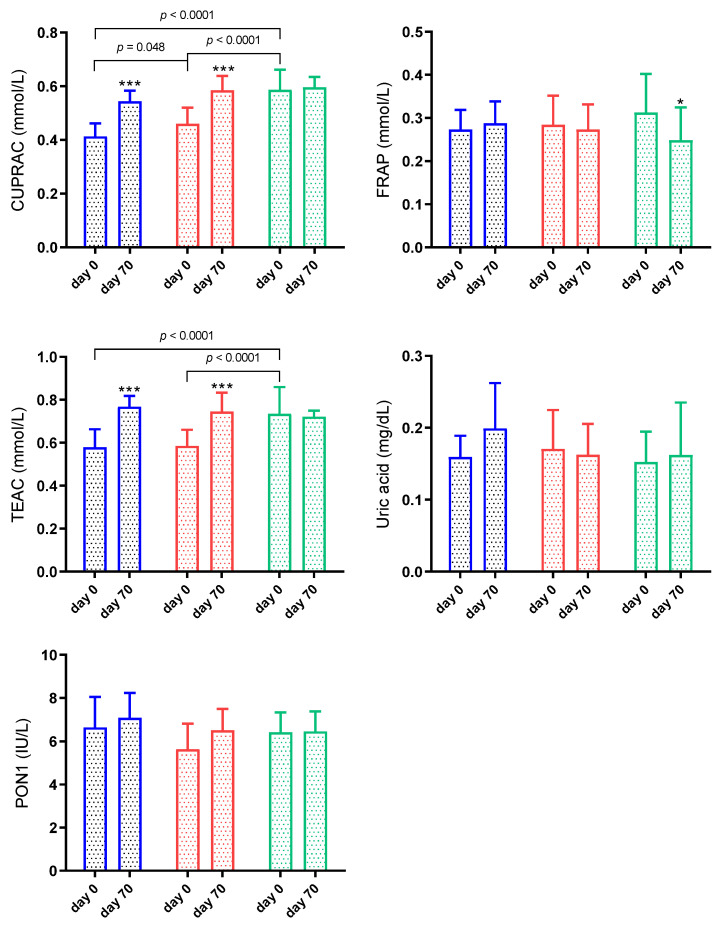
Results for cupric reducing antioxidant capacity (CUPRAC), ferric reducing ability of plasma (FRAP), Trolox equivalent antioxidant capacity (TEAC), uric acid and paraoxonase 1 (PON1) in sheep naturally infected by gastrointestinal nematodes. G1 (blue bar): faecal worm egg counts (EPG) higher than 5000 and packed cell volume (PCV) lower than 24%; G2 (red bar): EPG higher than 5000 and PCV higher than 24%; G3 (green bar): EPG lower than 5000 and PCV higher than 24%. Asterisks indicate significantly different from day 0. * *p* < 0.05; *** *p* < 0.001.

At the first sampling, when the antioxidants were compared between the groups, CUPRAC and TEAC showed significantly lower concentrations in G1 and G2 than in G3 (*p* < 0.001). CUPRAC was also lower (*p* < 0.05) in G1 than G2. FRAP, uric acid and PON1 did not show significant changes between groups (*p* > 0.05) (Figure 3). After treatment, CUPRAC and TEAC increased significantly in G1 and G2 (*p* < 0.001) (Figure 3). FRAP decreased significantly (*p* < 0.05) at day 70 of G3. Uric acid and PON1 did not show significant changes between days (*p* > 0.05) (Figure 3). Significant interaction between time and group was observed in CUPRAC (*p* = 0.0005) and TEAC (*p* = 0.0001). On the other hand, there was no significant interaction between time and group in FRAP (*p* = 0.073), uric acid (*p* = 0.199) and PON1 (*p* = 0.305).

### 3.4. Oxidant Biomarkers 

**Figure 4:**
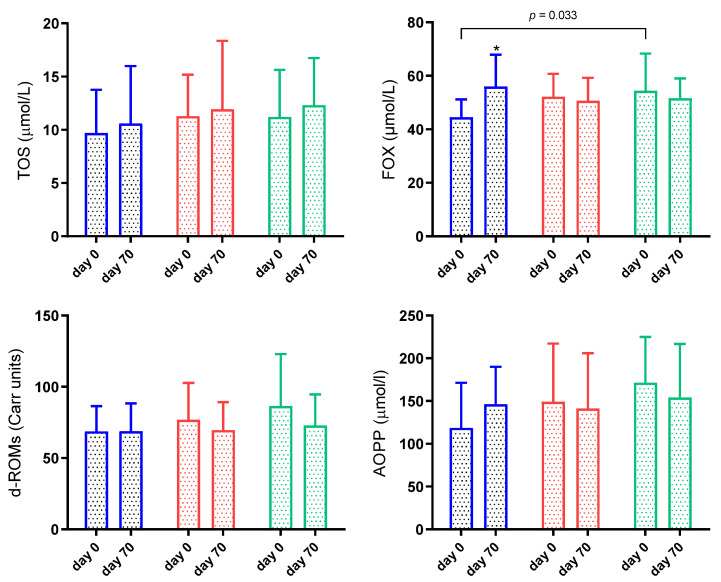
Results for total oxidant status (TOS), ferrous oxidation-xylenol orange (FOX), reactive oxygen metabolites derived compounds (d-ROMs), advanced oxidation protein products (AOPP) in sheep naturally infected by gastrointestinal nematodes. G1 (blue bar): faecal worm egg counts (EPG) higher than 5000 and packed cell volume (PCV) lower than 24%; G2 (red bar): EPG higher than 5000 and PCV higher than 24%; G3 (green bar): EPG lower than 5000 and PCV higher than 24%. Asterisks indicate significantly different from day 0. * *p* < 0.05.

Results for TOS, FOX, d-ROMs and AOPP are shown in Figure 4. FOX on day 0 was significantly lower in G1 than G3 (*p* = 0.03) and increased significantly (*p* < 0.05) in G1 on day 70. TOS, d-ROMs and AOPP showed no significant changes throughout the study (*p* > 0.05) (Figure 4).

There was a significant interaction between time and group in FOX (*p* = 0.018). There was no significant interaction between time and group in TOS (*p* = 0.987), d-ROMs (*p* = 0.568) and AOPP (*p* = 0.356).

### 3.5. Inflammatory Biomarkers 

**Figure 5:**
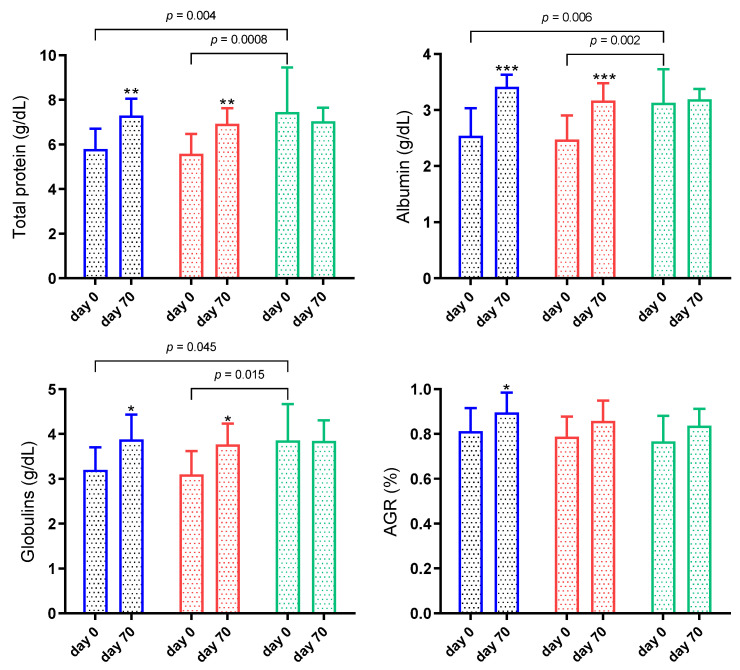
Total protein, albumin, globulin and albumin/globulin (AGR) ratio in sheep naturally infected by gastrointestinal nematodes and inserted in an integrated crop-livestock (ICL) system for 70 days. G1 (blue bar): faecal worm egg counts (EPG) higher than 5000 and packed cell volume (PCV) lower than 24%; G2 (red bar): EPG higher than 5000 and PCV higher than 24%; G3 (green bar): EPG lower than 5000 and PCV higher than 24%. Asterisks indicate significantly different from day 0. * *p* < 0.05; ** *p* < 0.01; *** *p* < 0.001.

The results for inflammatory biomarkers in sheep infected by gastrointestinal nematodes are shown in Figure 5. When the groups were compared in the first sampling, total proteins, albumin, and globulins concentrations were lower in G1 and G2 than G3 (*p* < 0.05). The Hp and BChE results in serum were below the detection limit of the assays for all groups (data not shown). 

When treatment was evaluated, sheep from G1 and G2 showed increased total protein, albumin and globulins concentrations (*p* < 0.05) at day 70. The AGR was also higher on day 70 in G1 (*p* < 0.05) (Figure 5). 

A significant interaction was observed in total proteins (*p* = 0.008) and albumin (*p* = 0.004). On the other hand, there was no significant interaction between time and group in globulins (*p* = 0.074) and AGR (*p* = 0.683).

### 3.6. Correlation Study

Table 1 shows Spearman correlation coefficients and significance between EPG and all biomarkers studied in the sheep with gastrointestinal nematodes during the 70 days of the ICL system. Regarding thiol-disulphide homeostasis, the analytes that showed the highest correlation coefficients with EPG were TT, SH, and SH/TT ratio, which correlated negatively (*r* > −0.72), and with SS/TT, SS/SH ratios that correlated positively (*r* > 0.71). Within antioxidant markers, the highest correlation with EPG was between TEAC (*r* = −0.68) and CUPRAC (*r* = −0.66). Among the inflammatory biomarkers, the highest coefficient correlation was found negatively between EPG and albumin and total proteins (*r* = −0.69).

## 4. Discussion

The study of thiol-disulphide homeostasis for the clinical evaluation of oxidative stress in different diseases is gaining interest in recent years, especially in human medicine [45,46]. The analytes used for the assessment of thiol-disulphide homeostasis are total thiol (TT), native thiol (SH) and disulphides (SS). The TT is integrated mainly by free cysteine, glutathione, cysteine residues and albumin and includes both reduced and oxidised thiols, whereas SH represents only the reduced thiol [47]. The thiols have a high vulnerability to oxidation [45], forming SS when oxidised [48,49]. Since albumin is the main component of the plasma thiol pool, the SS/TT, SS/SH and SH/TT ratios are used to avoid the possible effect of albumin [25]. 

In this study, the thiol-disulphide homeostasis system changed in sheep naturally infected by gastrointestinal nematodes, and in most analytes, this change was correlated with the parasite load. This correlation was negative for TT, SH, SS and SH/TT ratio and positive for SS/TT and SS/SH ratios. Furthermore, the animals infected with more than 5000 EPG, independent of having anaemia or not, showed lower TT and SH than animals having lower than 5000 EPG. This may indicate that the infection by gastrointestinal nematodes in sheep causes changes in the steady-state balance between reduced and oxidised thiol. These changes consist in the decrease in the levels of reduced forms and increasing the oxidised components and consequently the ratios among SS and thiol forms, suggesting a state of oxidative stress. In addition, the results reveal that the more intense the parasite burden the greater the oxidative response. Similar changes have been described in sheep with sarcoptic mange [26] and in other species and parasitic diseases such as cutaneous leishmaniasis in humans [50].

This report also evaluated the changes after treatment in thiol-disulphide metabolism in sheep infected by gastrointestinal nematodes. Two different treatment methods were used in the animals of our study: anthelmintic closantel with ICL and only ICL. Although the practice of only using anthelmintics was common in the past, it is not sustainable and has led to resistance [51,52]. Therefore, an integrated approach (ICL) including environmental management and chemoprophylaxis to minimise the pressure for parasite adaptation and parasite control is frequently used nowadays [31]. In our study, the two different treatment methods used were efficient in decreasing parasite load and improving inflammation and oxidative status of the sheep. Our results are in line with a previous study in which the ICL associated with a good nutrition plan resulted in progressively declining degrees of gastrointestinal parasite infections and satisfactory performance of lambs [12].

The improvement in the oxidative status after the treatment was reflected in the changes in the dynamic thiol-disulphide homeostasis since all animals showed a reduced SS/TT ratio after treatment. Those highly infected also exhibited decreased SS/SH ratio at the end of the trial. Furthermore, all of them showed increased TT and SH. In this context, decreased SS/TT and SS/SH levels suggest that the rate of thiol being oxidised has declined, and the redox state of the thiol-disulphide system has been re-established. 

When other analytes regarding oxidative stress were evaluated, CUPRAC and TEAC were also decreased in both groups of sheep with high parasite load on the day of diagnosis. In addition, both biomarkers increased at the end of the trial just as the parasite burden reduced. TEAC was decreased in sheep infected with *H. contortus* and *T. circumcincta* [18,53,54]. In this study, FRAP did not change between groups; however, it increased after 70 days of study in G3, which could be in line with a previous study [19] that related its elevation to cellular protection during the infection with *H. contortus* in lambs. The antioxidant system has a cellular protective action against oxidative stress of cells, organs and tissue damage that results from parasitic invasion [55]. The depletion of systemic antioxidant status may reflect the increase in oxidative stress, which in this study was more pronounced in G1 and G2. The presence of the parasite increases mucosal damage and local oxidative stress, which depending on the intensity may lead to systemic changes. In addition, the pathogenesis of most parasitic infections has been associated with lipid peroxidation [56]. The increase in FOX in G1 after the treatment could be related to possible oxidative damage caused during the infection. However, additional studies are necessary to confirm this result. 

In general, according to our results, just CUPRAC, TEAC and the dynamic thiol-disulphide homeostasis could be used as markers of oxidative stress, which is related to the parasite burden, and monitor treatment of sheep infected by gastrointestinal nematodes. However, the biomarkers of thiol-disulphide homeostasis such as TT, SH and SS/TT and SS/SH ratios, concurrently with CUPRAC, showed more significant changes between groups and during treatments and may indicate that they could be more sensitive oxidative stress biomarkers for the monitoring of the response to treatment against gastrointestinal parasitism in sheep.

It is important to point out that the biomarkers of oxidative stress are nonspecific and cannot be used for diagnosis. Moreover, in agreement with a previous study [57], the use of various biomarkers should be strongly recommended for the evaluation of the redox balance in livestock species, in which reference intervals for each assay are still lacking. Additionally, the results of this study should be confirmed using a higher number of animals, and ideally, a healthy control group involving animals without parasites as well as a group of animals with more severe clinical signs of the disease should be included.

## 5. Conclusions

In our study conditions, the thiol-disulphide balance was impaired in lambs infected by gastrointestinal nematodes. The highly infected lambs showed lower TT and SH than those with a lower parasite burden. After treatment, these markers increased significantly in all groups of lambs, but the observed decreased SS/TT and SS/SH ratios were more intense in those highly infected animals. The results also indicated that the oxidised form of thiol decreased after reducing parasite load, where the thiol-disulphide homeostasis was more sensitive than other oxidative stress biomarkers in monitoring treatment. These results show that thiol-disulphide homeostasis is involved in parasitism by gastrointestinal nematodes in sheep and there is potential for using the biomarkers of this system to evaluate the severity of the disease and monitor treatment in sheep infected by gastrointestinal nematodes.

## Figures and Tables

**Table 1:** Spearman correlation coefficients and significance between the different biomarkers studied and the faecal worm egg counts (EPG) in lambs infected by gastrointestinal nematodes inserted in an integrated crop-livestock (ICL) system for 70 days.

Biomarker	Spearman Coefficient
PCV	−0.719 ***
TT	−0.756 ***
SH	−0.747 ***
SS	−0.293 *
SS/TT ratio	0.710 ***
SS/SH ratio	0.716 ***
SH/TT ratio	−0.721 ***
CUPRAC	−0.661 ***
FRAP	0.029
TEAC	−0.686 ***
Uric acid	0.070
PON1	−0.197
TOS	−0.071
FOX	−0.146
d-ROMs	0.100
AOPP	−0.187
Total proteins	−0.692 ***
Albumin	−0.693 ***
Globulin	−0.586 ***
AGR	−0.194

* *p* < 0.05; *** *p* < 0.001.
